# Functional Analysis of *GbFLS1045* Regulating the Metabolism of Flavonoids in *Ginkgo biloba* L.

**DOI:** 10.3390/metabo16030193

**Published:** 2026-03-13

**Authors:** Xiaojing Kang, Xuefei Xu, Dan Liu, Yizeng Lu, Chenliang Zhao, Limin Sun

**Affiliations:** 1 Key Laboratory of National Forestry and Grassland Administration for Warm Temperate Forest Ecosystem Conservation and Restoration, Forestry College of Shandong Agricultural University, Tai’an 271002, China; 2 Shandong Provincial Center for Forest and Grassland Germplasm Resources, Jinan 250102, China

**Keywords:** *Ginkgo biloba*, flavonoids, flavonol synthase, *GbFLS1045*, function analysis

## Abstract

**Objectives**: Flavonoids are a class of widely distributed secondary metabolites in plants. *Ginkgo biloba* leaves are rich in flavonoids and thus are utilized for extracting medicinal components to treat and prevent cardiovascular and cerebrovascular diseases. Flavonol synthase (FLS) serves as a key enzyme in the flavonol metabolic pathway. Numerous studies have identified and characterized FLS family genes across various plant species, all of which play crucial roles in regulating the flavonoid biosynthetic pathway. **Methods**: We measured the flavonoid content in *Ginkgo biloba* leaves across different months, performed transcriptomic analysis on leaves from months showing an increasing trend, and screened out the *GbFLS1045* gene involved in the synthesis of the FLS enzyme. Molecular biology techniques were then employed to explore the function of the *GbFLS1045* gene. **Results**: From June to August, the flavonoid content in *Ginkgo biloba* leaves exhibited an upward trend, and we found that *GbFLS1045* is localized in the cytoplasm, cell membrane, and nucleus through transient transformation in *Nicotiana tabacum*. Overexpression(OE) of *GbFLS1045* in *Arabidopsis thaliana* resulted in significantly higher levels of total flavonol glycosides, kaempferol, quercetin, and isorhamnetin in OE transgenic plants compared to WT controls. Furthermore, in OE lines of *Ginkgo biloba* callus, the isorhamnetin content was consistently elevated relative to both WT and Anti lines. **Conclusions**: *GbFLS1045* positively regulates flavonoid synthesis in *Ginkgo biloba*.

## 1. Introduction

Flavonoids are a class of low-molecular-weight polyphenolic compounds derived from the phenylpropanoid pathway, characterized by a basic C6–C3–C6 skeleton [1,2]. More than 10,000 flavonoids compounds have been identified to date, which can be classified into several major subgroups based on their specific structures, including flavanols, flavanones, flavonols, isoflavones, flavones, and anthocyanins [3]. As ubiquitous secondary metabolites present in various plant organs, flavonoids play crucial roles in plant growth and development, such as conferring resistance to abiotic and biotic stresses [4,5], influencing flower pigmentation [6], and regulating seed germination and root growth [7]. Moreover, flavonoids have been widely applied in the pharmaceutical industry due to their significant therapeutic potential, particularly in the prevention and treatment of cardiovascular and cerebrovascular diseases [8,9].

FLS serves as a key enzyme in the flavonol metabolic pathway. It competes with Dihydroflavonol4-reductase (DFR) for the common substrate, dihydroflavonol, thereby influencing the competitiveness of the flavonol synthesis branch for metabolic flux and ultimately affecting flavonol accumulation [10]. The first cDNA of the FLS family was cloned from *Petunia hybrida* [11] and its gene function was subsequently validated in both yeast and plants [12]. Since then, FLS family genes have been identified and functionally characterized in a wide range of plant species, including *Arabidopsis thaliana* [13,14,15], *Ginkgo biloba* [16], *Zea mays* [17], *Glycine max* [18], *Allium cepa* [19], *Camellia japonica* [20], *Cyclamen persicum* [21], and *Litchi chinensis* [22].

Research on FLS has been relatively extensive in *Arabidopsis thaliana*. To date, six FLS family genes, namely *FLS1*~*FLS6*, have been identified in the *Arabidopsis thaliana* genome [23]. Among these, *FLS1* is considered the most functionally critical, followed by *FLS3*, whereas the enzymatic activities encoded by other *FLS* genes are relatively low or even non-functional [13,14,24]. When Zhou et al. [20] introduced *CnFLS1* from *Camellia japonica* into *Nicotiana tabacum*, they observed a significant increase in flavonol content but a sharp decrease in anthocyanin levels in the transgenic plants. Similarly, Han et al. [25] found that introducing antisense strands of *FLS* genes from *Zea mays* and *Helianthus annuus* into *Nicotiana tabacum* and *Petunia hybrida*, respectively, led to a marked increase in anthocyanin content and a concurrent decrease in flavonol levels in the flowers of transgenic plants. Furthermore, overexpression of FLS family genes from *Prunus persica* [26], *Rosa rugosa* [26], *Camellia sinensis* [27], and *Chrysanthemum* × *morifolium* [28] in *Nicotiana tabacum* resulted in lighter petal coloration, significantly increased flavonol accumulation, and a substantial reduction in anthocyanin content. Clearly, genes of the FLS family play a crucial regulatory role in flavonol accumulation in plants. This, in turn, profoundly influences the metabolic partitioning of the anthocyanin biosynthesis pathway and serves as an important determinant in the modulation of plant coloration [29].

*Ginkgo biloba* leaves are rich in flavonoids, which have consistently served as a key driver in the development of natural product-based pharmaceuticals due to their broad spectrum of physiological and pharmacological activities [30]. As a core regulator of the flavonol metabolic network, FLS has been extensively identified and studied in numerous plant species, with its gene family members demonstrated to play important roles in modulating the flavonoid biosynthetic pathway. In the present study, through dynamic quantification of flavonoid contents in *Ginkgo biloba* leaves across different months combined with transcriptome sequencing analysis, we identified the candidate gene *GbFLS1045*. Its function within the flavonoid biosynthetic pathway was further validated using molecular biology experiments.

## 2. Materials and Methods

### 2.1. Plant Materials and Treatments

From April to November, on the 15th of each month, sufficient leaf samples were randomly collected from three individual *Ginkgo biloba* ‘TS10’ family trees preserved in the Jujube Garden Conservation Repository at the Shandong Provincial Center for Forest and Grass Germplasm Resources in Jinan, Shandong Province, China (36°25′ N–37°09′ N, 117°10′ E–117°35′ E; continental climate within the warm temperate monsoon zone). These samples were used to determine the flavonoid content in *Ginkgo biloba* leaves at different developmental stages.

Seeds collected from the maternal tree of the TS10 (an ancient *Ginkgo biloba* tree located at Laojuntang, Tai’an City, Shandong Province, China; 36°12′ N, 117°11′ E) were washed, and their embryos were aseptically excised under sterile conditions. The embryos were then inoculated onto MS medium and cultured in an illuminated incubator under the following conditions: light intensity of 2000 lx, temperature of 25 °C, and a photoperiod of 16 h light/8 h darkness, to generate the sterile seedlings used in the experiments. Subsequently, leaf explants from these sterile seedlings were cultured under the same incubator conditions to induce callus formation. The callus induction medium consisted of MS basal medium supplemented with NAA (1-Naphthaleneacetic acid: 4 mg·L^−1^) and KT (Kinetin: 2 mg·L^−1^).

The experimental materials included *Arabidopsis thaliana* (ecotype Columbia) and *Nicotiana benthamiana*. Seeds were germinated and grown on MS medium for approximately 8 days. The resulting seedlings were then transplanted into soil and cultivated in a controlled environment chamber under the following conditions: light intensity of 120 µmoL m^−2^ s^−1^, temperature of 20–24 °C a 16 h light/8 h dark photoperiod, and 50% relative humidity. Watering was performed twice per week.

### 2.2. Determination of Flavonoid Content

The flavonoid content in *Ginkgo biloba* leaves from different periods was determined using the G0118F assay kit form Suzhou Grace Biotechnology Co., Ltd. (Suzhou 215000, China). Based on the NaNO_2_-Al(NO_3_)_3_-NaOH colorimetric method, with absorbance measured at 510 nm. For transgenic materials, flavonoid content was analyzed by liquid chromatography [31]. Briefly, the contents of individual flavonols (including kaempferol, quercetin, and isorhamnetin) and total flavonol glycosides were determined following the method described by Zhao et al. [31] with slight modifications. Frozen leaf tissue (120 mg) was ground and homogenized with 1.5 mL of 80% methanol, ultrasonicated for 30 min, and then extracted at 4 °C for 16 h. After centrifugation, the supernatant was concentrated, reconstituted in 80% methanol, and filtered through a 0.22 μm membrane prior to analysis. Total flavonol glycoside content was calculated as the sum of the three aglycone concentrations multiplied by a conversion factor of 2.51.

### 2.3. RNA Transcriptome Sequencing

Based on the results of flavonoid content measurements, leaf samples from *Ginkgo biloba* individuals with the highest (H) and lowest (L) flavonoid levels were selected from June (3), July (2), and August (1), during which flavonoid content showed a steady increase. Three biological replicates were included per group, resulting in a total of six libraries (H3, L3, H2, L2, H1, L1). Library construction and sequencing were performed following the same methods as described in a previous study [32]. Filtered reads were aligned to the *Ginkgo biloba* reference genome “https://ngdc.cncb.ac.cn/gwh/Assembly/18742/show (accessed on 1 August 2024)”. Genes with |log_2_ Fold Change| ≥ 1 and padj < 0.05 were identified as DEGs. Expression levels of DEGs were quantified as fragments per kilobase of transcript per million mapped reads (FPKM). Subsequent GO enrichment analysis and KEGG pathway enrichment analysis were conducted using the same analytical approaches as reported by Li et al. [32].

### 2.4. qRT-PCR

Total RNA was extracted using VeZol Reagent from Vazyme Biotech Co., Ltd. (Nanjing 210000, China) according to the manufacturer’s instructions. Following the method described by Zhao et al. [31], the *GAPDH* gene of *Ginkgo biloba* was selected as the internal reference gene. Primers were designed using Primer 5 (Supplementary Table S1), and quantitative reverse transcription-PCR (qRT-PCR) was subsequently performed.

### 2.5. Subcellular Localization

The CDS sequence of *GbFLS1045* without the termination codon was ligated into the pCAMBIA 1300 vector. Subsequently, *Agrobacterium* GV 3101 harboring the 35S::*GbFLS1045*-GFP construct was cultivated at 28 °C with shaking at 200 rpm for 12 h, followed by centrifugation at 4000× *g* rpm for 10 min to collect the bacterial cells. The pellet was resuspended in an infiltration buffer containing 10 mM MgCl_2_, 10 mM MES, and 150 µM acetosyringone to an OD600 of 1. The suspension was then injected into the abaxial side of tobacco leaves using a needleless syringe. After 48 h of incubation in the dark, the lower epidermis of the infiltrated leaves was carefully peeled and examined for GFP fluorescence using a laser scanning confocal microscope (LSM880; Zeiss, Oberkochen, Germany).

### 2.6. Preparation of Transgenic Materials

The CDS sequence of *GbFLS1045* was ligated into the pPZP211 vector to construct the overexpression vector, while the antisense CDS sequence of *GbFLS1045* was ligated into the pPZP 211 vector to construct the suppression vector, and the plasmids were introduced into *Agrobacterium* GV 3101. The inflorescences of *Arabidopsis thaliana* were infected with *Agrobacterium* containing the overexpression vector, and after seed maturation, successfully transformed *Arabidopsis thaliana* seeds (T0) were selected on MS medium containing 50 µg/mL kanamycin. The *Arabidopsis thaliana* plants were then cultured and screened to the T2 generation, and their leaves were flash-frozen in liquid nitrogen and stored at −80 °C for subsequent experiments. *Agrobacterium* containing the overexpression vector and *Agrobacterium* containing the suppression vector were respectively resuspended in MS resuspension solution containing 100 μM As acetosyringone to an OD600 of 0.6–0.8, and callus tissues were immersed in the suspension for 15 min. The callus tissues were then placed on MS medium containing 100 μM As acetosyringone and dark-cultured for 3 days, after which samples were collected, flash-frozen in liquid nitrogen, and stored at −80 °C for subsequent experiments. Sterile *Ginkgo biloba* seedlings were immersed in *Agrobacterium* infection solutions containing either the overexpression vector or the suppression vector, subjected to continuous vacuum infiltration for 20 min using a vacuum pump, and then placed on MS medium. After 1 day of dark culture followed by 3 days of light culture, their leaves were collected, flash-frozen in liquid nitrogen, and stored at −80 °C for subsequent experiments.

## 3. Results

### 3.1. Seasonal Variation in Flavonoid Content in Ginkgo biloba Leaves

**Figure 1:**
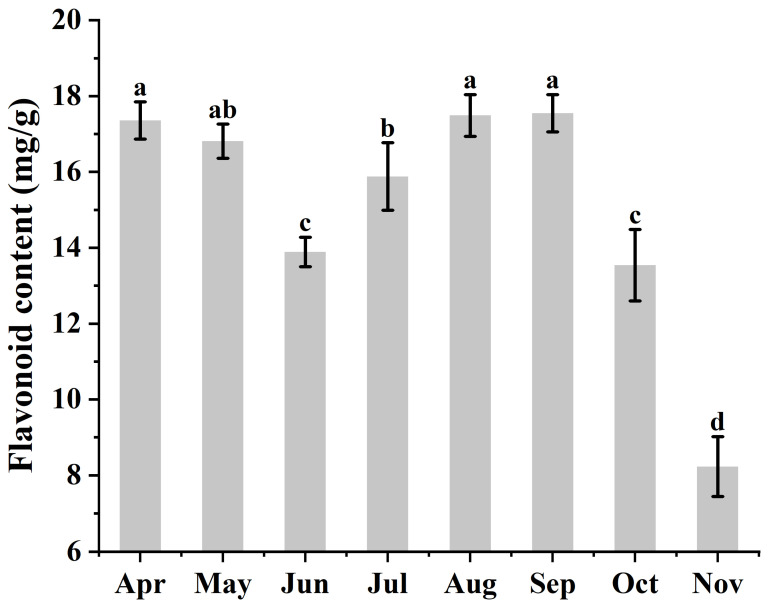
Flavonoid variation in *Ginkgo biloba* leaves during 2023. *X*-axis: Different seasonal groups; *Y*-axis: Mean flavonoid content in *Ginkgo biloba* leaves across different seasons; Error bars: Mean + SD; Letters in the figure: Different letters on the bars indicate a significant difference between groups for the corresponding compound, while the same letter indicates no significant difference. The letters themselves have no inherent meaning.

It was found by D-test that the flavonoid content in *Ginkgo biloba* leaves exhibited highly significant seasonal variation (*p* < 0.01). In early leaf development (April), the flavonoid content was relatively high (17.38 mg·g^−1^). Subsequently, from May to June, as leaves continued to grow, the flavonoid content gradually decreased to 16.81 mg·g^−1^ and 13.89 mg·g^−1^, respectively. Starting in June, the flavonoid content began to increase steadily, reaching 17.49 mg·g^−1^ in August and peaking at 17.55 mg·g^−1^ in September. After the leaves turned yellow in autumn, the flavonoid content dropped sharply, reaching a very low value (8.23 mg·g^−1^) around leaf fall in November (Figure 1).

### 3.2. Transcriptome Sequencing Analysis of Ginkgo biloba Leaves at Different Growth Stages

**Figure 2:**
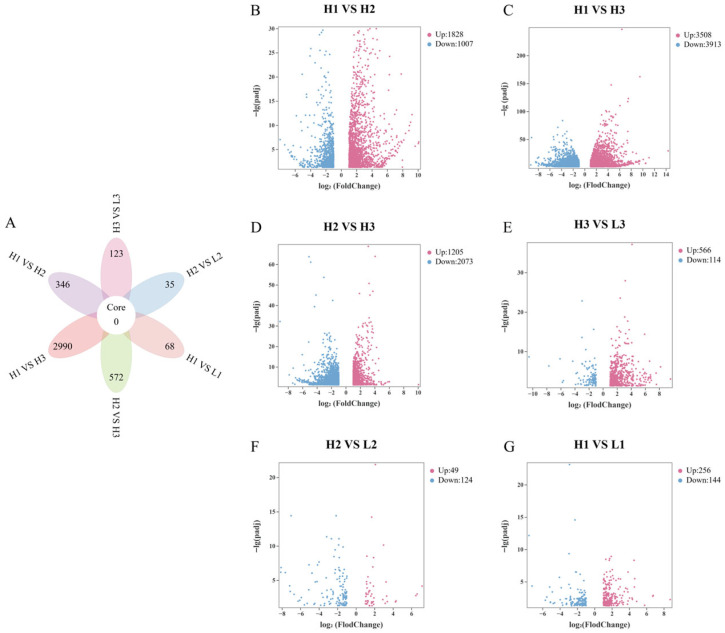
(**A**) The six sets of Venn diagrams showing DEGs. The central number indicates the overlapping DEGs among all groups, and the numbers in the petals indicate the unique DEGs for each group. (**B**–**G**) Volcano plots showing DEGs for each group.

Following filtration of raw reads obtained from Illumina HiSeq™ 4000 sequencing, high-quality Clean Reads were obtained, accounting for 92.70% to 98.58% of the total. Transcriptome sequencing analysis yielded approximately 40.46–47.86 million high-quality reads per sample, with 79.52% to 91.53% successfully mapped to the reference genome (Supplementary Table S2). Using the thresholds of |log_2_ Fold Change| ≥ 1 and padj < 0.05, the numbers of DEGs identified in each comparison group were as follows (Figure 2B–G): 2835 DEGs (1828 upregulated and 1007 downregulated) for H1 vs. H2; 7421 DEGs (3508 upregulated and 3913 downregulated) for H1 vs. H3; 3278 DEGs (1205 upregulated and 2073 downregulated) for H2 vs. H3; 400 DEGs (256 upregulated and 144 downregulated) for H1 vs. L1; 173 DEGs (49 upregulated and 124 downregulated) for H2 vs. L2; and 680 DEGs (566 upregulated and 114 downregulated) for H3 vs. L3. A certain number of unique DEGs, defined as genes that were significantly differentially expressed only in a specific pairwise comparison, were identified in each comparison group (Figure 2A). The H1 vs. H3 comparison contained the highest number of unique DEGs (2990), while the H2 vs. L2 comparison contained the fewest (only 35). The counts of unique DEGs for the other groups were as follows: 346 for H1 vs. H2, 572 for H2 vs. H3, 68 for H1 vs. L1, and 123 for H3 vs. L3.

### 3.3. Enrichment Analysis of Differentially Expressed Genes in Ginkgo biloba Leaves at Different Growth Stages

**Figure 3:**
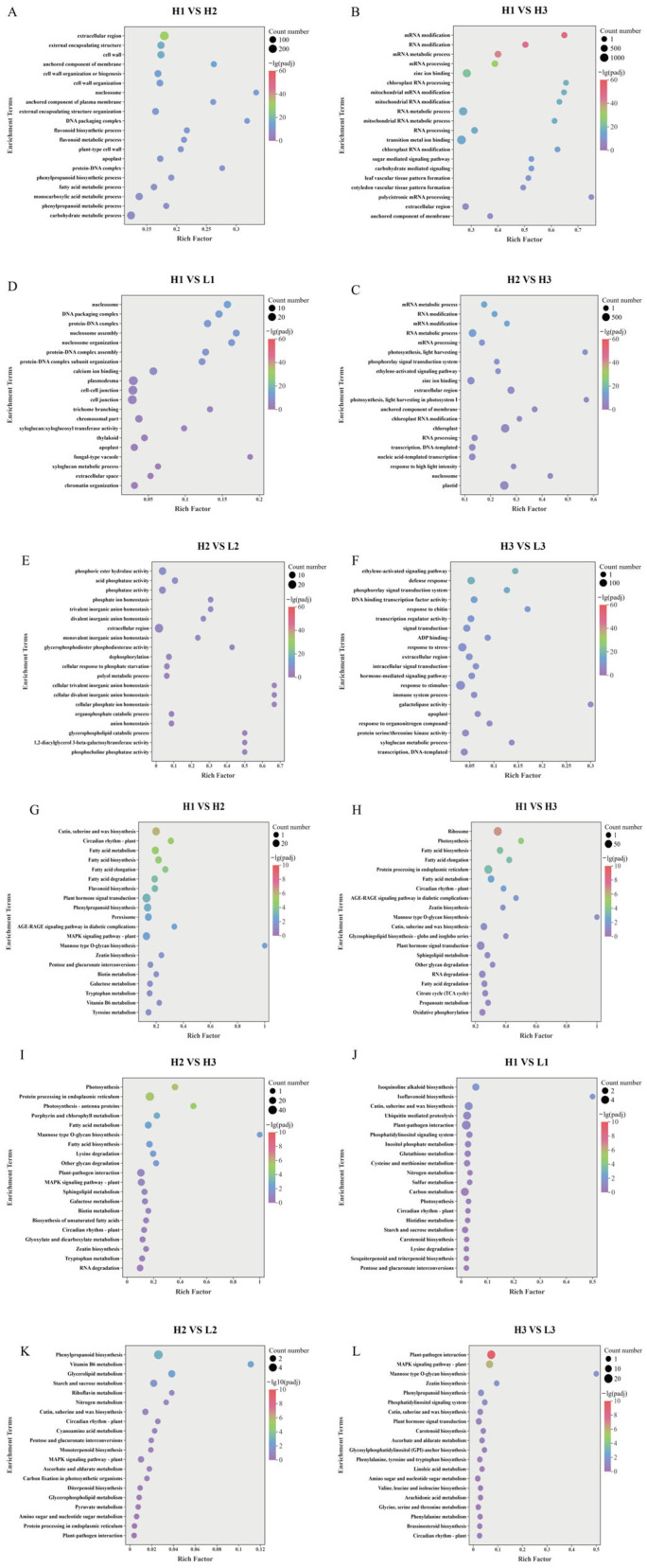
(**A**–**F**) GO enrichment analysis of DEGs for each group. *X*-axis: Rich factor; *Y*-axis: GO pathway name. (**G**–**L**) KEGG pathway enrichment of DEGs for each group. *X*-axis: Rich factor; *Y*-axis: KEGG pathway name. Bubble color reflects the padj; Bubble size: The number of genes enriched in the corresponding pathway. Rich factor: The enrichment factor, defined as the ratio of the number of differentially expressed genes annotated to a given pathway to the total number of genes annotated to that pathway.

To explore the biological functions of these DEGs, GO and KEGG enrichment analyses were performed. Among the top 20 enriched GO terms (Figure 3A–F), ‘polycistronic mRNA processing’ (Biological Process) was the most significantly enriched in the H1 vs. H3 comparison, ‘photosynthesis, light harvesting in photosystem I’ (Biological Process) was the most significantly enriched in H2 vs. H3, while ‘cellular phosphate ion homeostasis’,‘cellular divalent inorganic anion homeostasis’ and ‘cellular trivalent inorganic anion homeostasis’ (all Biological Process) were jointly ranked first in H2 vs. L2. For the Cellular Component category, ‘nucleosome’ was most significantly enriched in H1 vs. H2, and ‘fungal-type vacuole’ was most significantly enriched in H1 vs. L1. Regarding Molecular Function’ galactolipase activity’ was the most significantly enriched term in H3 vs. L3. In terms of the largest number of enriched genes, ‘external encapsulating structure organization’ (Biological Process) contained the most DEGs in H1 vs. H2, ‘transition metal ion binding’ (Molecular Function) had the highest count in H1 vs. H3, and ‘plastid’ (Cellular Component) contained the most DEGs in H2 vs. H3. For the H1 vs. L1 comparison, ‘plasmodesma’, ‘cell–cell junction’ and ‘cell junction’ (all Cellular Component) jointly had the highest number of enriched genes. ‘Extracellular region’ (Cellular Component) contained the most DEGs in H2 vs. L2, and ‘response to stimulus’ (Biological Process) had the highest count in H3 vs. L3. Among the top 20 enriched KEGG pathways (Figure 3G–L), ‘Mannose type O-glycan biosynthesis’ was the most significantly enriched in the H1 vs. H2, H1 vs. H3, H2 vs. H3, and H3 vs. L3 comparisons. In contrast, the most significantly enriched pathways were ‘Isoflavonoid biosynthesis’ for H1 vs. L1 and ‘Vitamin B6 metabolism’ for H2 vs. L2. Regarding the number of enriched genes, ‘Plant–pathogen interaction’ contained the largest number of DEGs in both H1 vs. L1 and H3 vs. L3. ‘Plant hormone signal transduction’ had the highest gene count in H1 vs. H2, ‘Ribosome’ in H1 vs. H3, ‘Protein processing in endoplasmic reticulum’ in H2 vs. H3, and ‘Phenylpropanoid biosynthesis’ in H2 vs. L2.

### 3.4. Screening and Analysis of Differentially Expressed Genes in the Flavonoid Biosynthesis Pathway of Ginkgo biloba Leaves at Different Growth Stages

**Figure 4:**
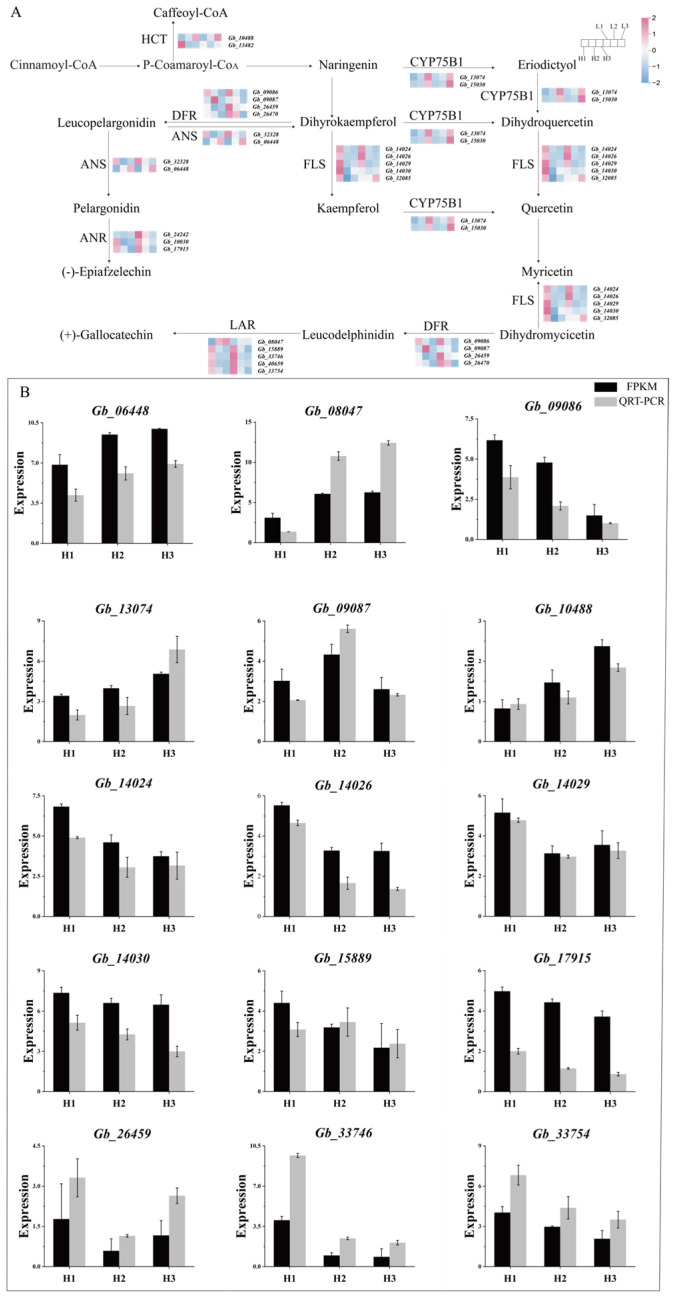
(**A**) Gene expression related to the flavonoid biosynthesis pathway. Red indicates upregulation, blue indicates downregulation. (**B**) qRT-PCR expression levels and FPKM values of 15 differential genes in the flavonoid biosynthesis pathway.

Based on the transcriptomic data, we further analyzed the regulatory patterns of DEGs within the flavonoid biosynthesis pathway (Figure 4A). The results revealed that 23 genes belonging to seven key enzymes were differentially expressed across various comparison groups, and the correlation between the expression levels of these relevant genes and the accumulation of major flavonoid compounds was examined (Supplementary Table S3). Notably, the FLS family genes *Gb14024*, *Gb14026* and *Gb14029*, which are directly involved in the biosynthesis of kaempferol and quercetin, exhibited higher expression levels in the H1 and L1 groups. Furthermore, qRT-PCR analysis of 15 genes, including *Gb14024*, *Gb14026* and *Gb14029*, confirmed that their expression trends were consistent with those observed in the transcriptome data (Figure 4B), thereby validating the reliability of the transcriptomic dataset. For the convenience of subsequent research, the gene *Gb14024* was designated as *GbFLS1045*.

### 3.5. Subcellular Localization of GbFLS1045

**Figure 5:**
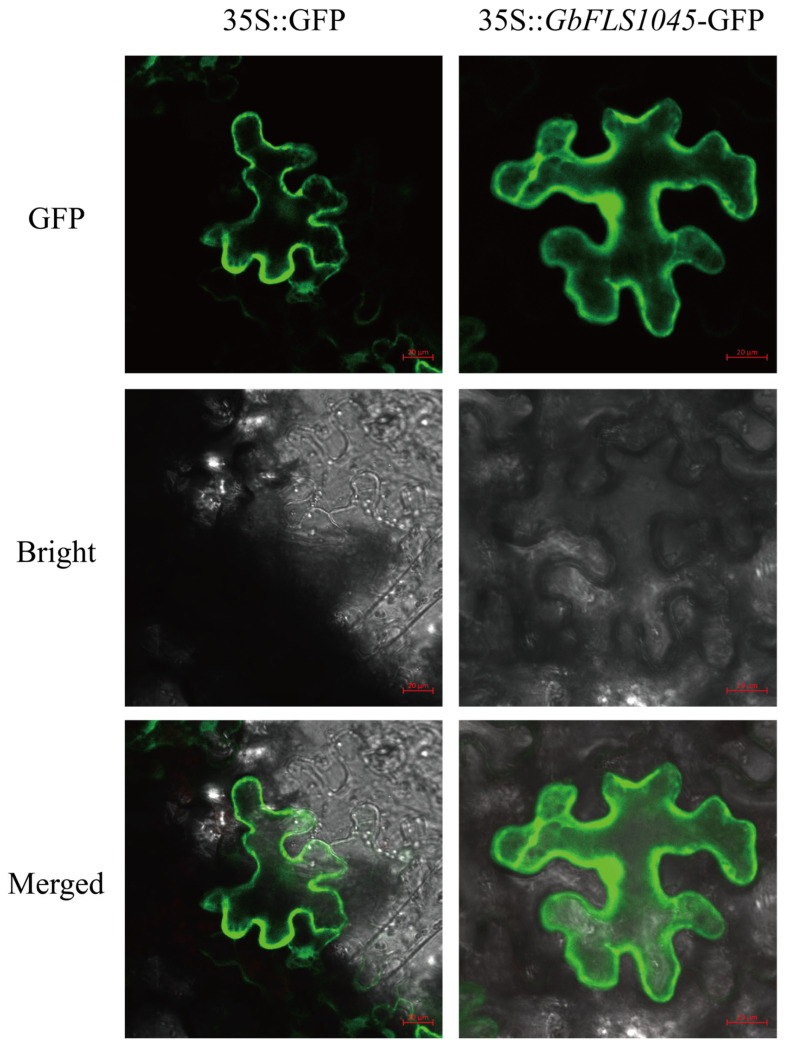
Subcellular Localization of *GbFLS1045*. 35S::GFP acted as negative control. Scale bar = 20 μm.

To further validate the subcellular functional localization of the GbFLS1045 gene, transient transformation was performed in *Nicotiana benthamiana* leaves via *Agrobacterium*-mediated infiltration. Observation of the lower epidermal cells using super-resolution confocal laser scanning microscopy revealed that the 35S::*GbFLS1045*-GFP fluorescence signals were distributed in the cell membrane and nucleus (Figure 5), indicating that the *GbFLS1045* gene can be expressed in the cell membrane and nucleus.

### 3.6. Overexpression of GbFLS1045 Promotes Flavonoid Accumulation in Arabidopsis thaliana

**Figure 6:**
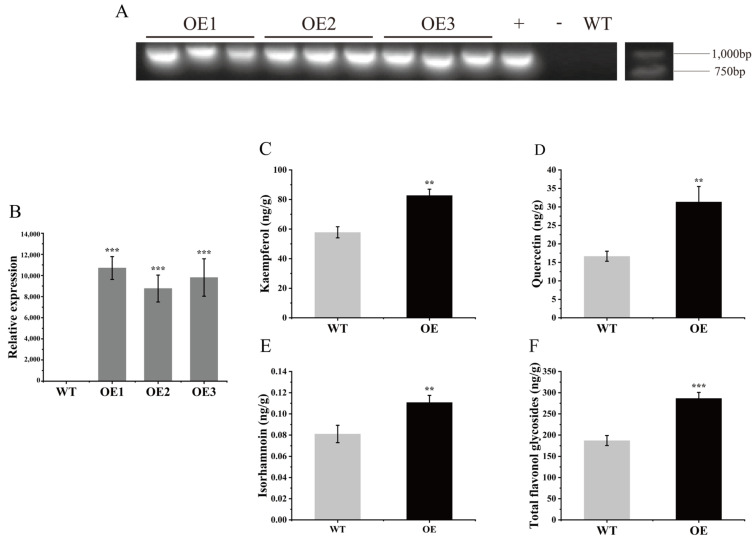
(**A**) Verification of positive transgenic *Arabidopsis thaliana* plants overexpressing *GbFLS1045* by PCR. “+” indicates positive control, “−” indicates negative control. (**B**–**F**) Expression level of *GbFLS1045* and contents of quercetin, kaempferol, isorhamnetin, and total flavonol glycosides in transgenic *Arabidopsis thaliana* lines. ** *p* ≤ 0.01 denotes significance compared to the control group; *** *p* ≤ 0.001 denotes significance compared to the control group.

To further validate the function of the GbFLS1045 gene in flavonoid biosynthesis, heterologous expression was performed via Agrobacterium-mediated transformation (Figure 6A). Subsequent quantification of flavonoid compounds revealed a significant increase in their contents in the leaves. Compared with the WT plants, the levels of kaempferol, quercetin, isorhamnetin, and total flavonol glycosides were elevated by 43.3%, 88.2%, 37.5%, and 53.2%, respectively (Figure 6B–F).

### 3.7. Transient Expression of GbFLS1045 in Ginkgo biloba Callus and Sterile Seedlings

**Figure 7:**
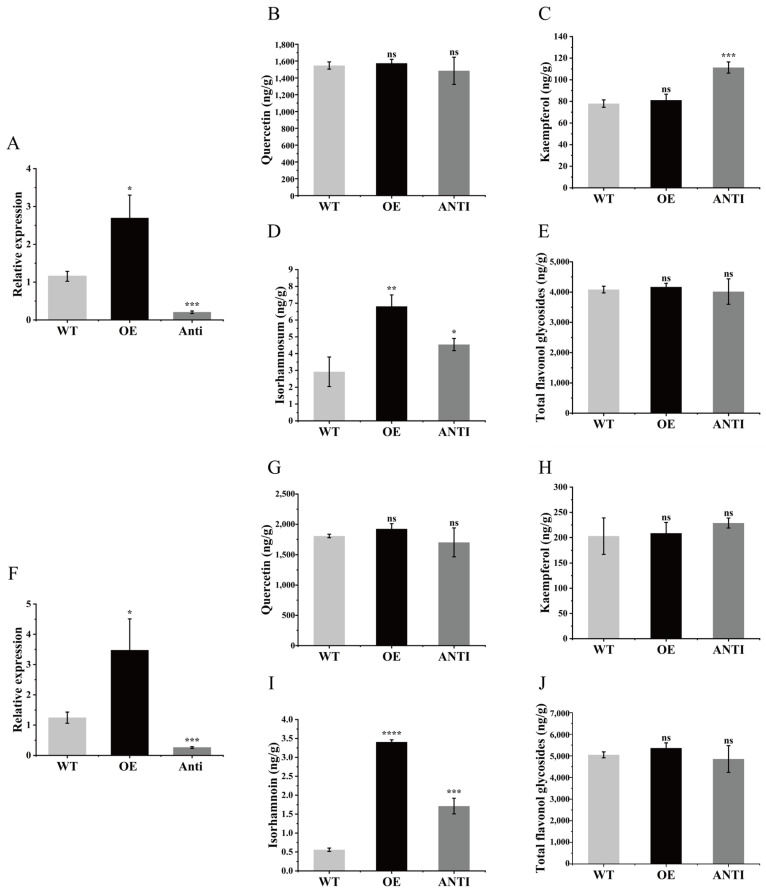
(**A**–**E**) Expression level of *GbFLS1045* and contents of quercetin, kaempferol, isorhamnetin, and total flavonol glycosides in transgenic *Ginkgo biloba* callus lines. (**F**–**J**) Expression level of *GbFLS1045* and contents of quercetin, kaempferol, isorhamnetin, and total flavonol glycosides in transgenic *Ginkgo biloba* sterile seedling lines. ns *p* > 0.05 denotes significance compared to the control group; * *p* ≤ 0.05 denotes significance compared to the control group; ** *p* ≤ 0.01 denotes significance compared to the control group; *** *p* ≤ 0.001 denotes significance compared to the control group; **** *p* ≤ 0.0001 denotes significance compared to the control group.

After overexpression and suppression of *GbFLS1045* in *Ginkgo biloba* callus and sterile seedlings, respectively, the expression level of *GbFLS1045* showed a significant increase and decrease compared with the WT (Figure 7A,F). In the overexpression lines of both callus and sterile seedlings, the contents of kaempferol, quercetin, and total flavonol glycosides exhibited no significant changes relative to WT, whereas the content of isorhamnetin was significantly elevated. Specifically, isorhamnetin content increased by 133% in overexpressing callus and by 509% in overexpressing sterile seedlings compared with WT. In contrast, in the suppression lines of both callus and sterile seedlings, the contents of all the aforementioned compounds showed no significant changes relative to WT (Figure 7B–E,G–J).

## 4. Discussion

The content of flavonoids in plant leaves exhibits dynamic seasonal variations, a phenomenon observed across various species. For instance, flavonoids in *Hippophae rhamnoides* leaves show significant changes from May to November, peaking in July [33]. Similarly, *Camellia sinensis* leaves display distinct flavonoid levels in April, June, August, September, and October [34]. In *Ginkgo biloba* leaves, flavonoid content reaches its highest level in May, declines to the lowest point in June, begins to recover to a relatively high level after July, and stabilizes in August [35]. In our study, measurements of flavonoid content in *Ginkgo biloba* leaves from April to October revealed relatively high levels in April, a slight decrease in May, and a decline to the lowest point in June, followed by a gradual recovery and stabilization in August. This trend aligns with the pattern reported by Guo et al. [35] for *Ginkgo biloba* leaf flavonoids from May to August. However, our subsequent measurements showed a rapid decrease in *Ginkgo biloba* leaf flavonoids in October. This decline may be attributed to the onset of autumn in northern China, where lower temperatures in October induce the expression of genes related to anthocyanin synthesis in *Ginkgo biloba* [36]. When metabolic flux is primarily directed toward the anthocyanin biosynthesis pathway, it can significantly interfere with the flavonoid biosynthesis branch, leading to reduced accumulation of flavonoids [27].

Based on the aforementioned results, we selected the period from June to August, during which flavonoid content in *Ginkgo biloba* leaves exhibited an increasing trend, for transcriptome sequencing. Through data analysis, we successfully identified the key gene *GbFLS1045* regulating the flavonoid biosynthesis pathway in *Ginkgo biloba* leaves. Previous studies have indicated that FLS is a pivotal branch point enzyme that competes with DFR for their common substrate (dihydroflavonol), thereby influencing the competitiveness of the flavonol synthesis branch for metabolic flux and subsequently affecting flavonol accumulation [10]. In the present study, we observed that overexpression of *GbFLS1045* in *Arabidopsis thaliana* leaves significantly increased the contents of kaempferol, quercetin, isorhamnetin, and total flavonol glycosides. This suggests that *GbFLS1045* overexpression enhanced the competitiveness of the flavonol synthesis branch for metabolic flux, thereby promoting flavonoid accumulation in *Arabidopsis thaliana*, which aligns with the findings of Jiang et al. [27]. However, in *Ginkgo biloba* calli and sterile seedlings, overexpression of *GbFLS1045* did not lead to significant changes in kaempferol, quercetin, or total flavonol glycoside contents. In contrast, the content of isorhamnetin, a downstream methylated derivative of quercetin, was markedly increased. This phenomenon may be attributed to the overall limitation of metabolic flux on the effect of single-gene regulation [37], where insufficient precursor supply or competition for metabolic flux towards other branches restricts the accumulation of primary products. The selective increase in isorhamnetin might result from the efficient and specific methylation of quercetin by abundantly present downstream methyltransferase enzymes in plants [38]. For instance, AaOMT identified from *Artemisia annua* was reported to efficiently catalyze the conversion of quercetin to its methylated derivatives [39]. Therefore, *GbFLS1045* overexpression may have synergistically enhanced the activity of specific gene families, facilitating the rapid conversion of newly synthesized quercetin into isorhamnetin. Furthermore, when the expression of *GbFLS1045* was suppressed in *Ginkgo biloba* calli and sterile seedlings, the contents of kaempferol, quercetin, and total flavonol glycosides showed no significant alterations. This could be due to the complex transcriptional network governing gene expression, where merely interfering with *GbFLS1045* allows its duplicate genes to provide functional compensation, thereby maintaining metabolic homeostasis. This is consistent with the observation in *Arabidopsis thaliana*, where multiple duplicate genes can compensate for the loss of a single gene through alternative pathways [40].

## 5. Conclusions

The flavonoid content in *Ginkgo biloba* leaves exhibits substantial seasonal variation, demonstrating highly significant differences across different seasons. In early leaf development during April, flavonoid content is relatively high. Subsequently, from May to June, as the leaves grow, the flavonoid content gradually declines. Beginning in June, the flavonoid content starts to increase progressively, reaching its peak in September. Following the yellowing of *Ginkgo biloba* leaves in autumn, the flavonoid content drops sharply, reaching very low levels around leaf abscission. Transcriptome analysis based on this pattern identified numerous differentially expressed genes involved in *Ginkgo biloba* flavonoid biosynthesis and led to the screening of the *GbFLS1045* gene. Overexpression of *GbFLS1045* in *Arabidopsis thaliana* significantly increased the contents of kaempferol, quercetin, isorhamnetin, and total flavonol glycosides. In *Ginkgo biloba* calli and sterile seedlings, overexpression of *GbFLS1045* significantly elevated isorhamnetin content, whereas suppression of GbFLS1045 expression markedly reduced it. In summary, the *GbFLS1045* gene positively regulates flavonoid biosynthesis in *Ginkgo biloba*.

## Data Availability

The original contributions presented in this study are included in the article. Further inquiries can be directed to the corresponding authors.

## Supplementary Materials

The following supporting information can be downloaded at: https://www.mdpi.com/article/10.3390/metabo16030193/s1, Table S1: Gene Primers Used for Cloning, Vector Construction, and qRT-PCR Analysis; Table S2: The comparison results of each sample cDNA library with the *Ginkgo biloba* reference genome; Table S3: The expression levels of some significantly expressed DEGs.
